# Testing policies during an epidemic: An economic analysis

**DOI:** 10.1371/journal.pone.0322292

**Published:** 2025-05-27

**Authors:** Francesco Flaviano Russo

**Affiliations:** Federico II University and CSEF, Via Cintia, Monte Sant’Angelo, 80126, Napoli, Italy; United Arab Emirates University Faculty of Science: UAE University College of Science, UNITED ARAB EMIRATES

## Abstract

I build a stochastic epidemiological model with production and endogenous responses to the epidemic to compare different testing policies to isolate and quarantine the infectious: voluntary tests, random screenings and contact tracing. To increase the number of screened individuals at given testing capacity, I also allow for the use of group testing. Contact tracing with group testing is the best testing policy unless in case of: very contagious diseases, socially dense countries, high test costs and limited testing capacity. The gains include a lower mortality, a smaller output loss, and lower peaks of infections and hospitalizations. I show that sophisticated tracing technologies are not needed to achieve these gains.

## Introduction

The ability to identify and quarantine the biggest possible number of infectious individuals is a fundamental asset in the fight against an epidemic, which can avoid the massive disruption of economic and social activities brought by lockdowns. However isolating the infectious could be a daunting task whenever infected, but otherwise asymptomatic, individuals could be pathogen carriers that spread the disease, as in the Covid-19 case. Absent the possibility to frequently screen the entire population, which could perfectly identify all of the infectious, but which is logistically and economically unfeasible, the problem is how to optimally design a testing policy targeted to a subset of the population only.

I propose a stochastic epidemiological model with production to study the comparative performance of three alternative testing policies: periodical random screenings, tests on a voluntary basis, and a contact tracing^1^ that consists in identifying and testing the individuals who matched socially with the discovered infected. The key feature of the model is the agents’ endogenous response to the observed progression of the epidemic, in a context where everything that is observed is a function of the implemented testing policy. The model nests five new elements within an otherwise standard Susceptible-Infected-Recovered (SIR) model (Kermack and McKendrick [32]; Allen [3])of the spread of a contagious disease: an optimal individual choice over getting tested, conditional on the evolution of the epidemic and on having or not symptoms; a detailed model of the contact tracing process, that accounts for the structure of the economy; the possibility of concurrent infections, with similar symptoms; an optimal choice over the participation to social activities, as a function of the observed disease prevalence; an upper bound to the number of tests that can be processed each period.

The assumption of a limited testing capacity implies that capacity investments are not feasible in the midst of a non-anticipated epidemic, for instance because it takes time to train new professionals to process the tests, or because the very specialized manufacturers of the lab equipment might not be able to meet the demand. There is however an alternative possibility to increase the number of screened individuals without increasing the short-run testing capacity: group testing. In its simplest version, group testing consists in collecting two samples from each individual, testing first samples in groups, and then processing second samples individually only in case of a positive test result for the group. It is well known, at least since the work by Dorfman [17], that this strategy yields a significant reduction in the number of tests needed to screen a population, while keeping the same quality of the original tests. Israel and the state of Nebraska are two examples of the use of group testing in the context of the Covid-19 pandemic. An additional contribution of the paper consists in modeling group testing within an epidemiological model under alternative testing policies.

Group testing works best in case of a small incidence of positive test results, since second samples are seldom processed, so the number of individuals that can be screened is inversely proportional to the ratio of positive tests results to tests performed. In the context of an epidemic, the bigger is the number of tests performed, the smaller is this ratio. There is therefore an important reinforcing effect that makes group testing particularly effective: by increasing the number of screened individuals, it lowers the ratio of positives test results to tests performed, which, in turn, allows for an even bigger testing capacity increase and so on.

The actual number of tests to perform with group testing is contingent on the chosen group size, which must decrease with the ratio of positives to tests and, therefore, frequently adjusted. The problem is that the positives to tests ratio is unknown before processing the tests, which means that it is necessary to continuously forecast it, potentially making group testing difficult to implement. To make group testing operational, I consider a simple adaptive forecast model, with the optimal group size set, each period, according to the positives to tests ratio observed one period before. A further contribution of the paper is to propose this implementation strategy for group testing during an epidemic.

I solve the model numerically to find the optimal choice of a policy-maker that minimizes a loss function defined over three targets: the number of deaths, the cumulative output loss and the cost of the tests. I show that, for given pathogen characteristics, the optimal policy choice depends on the testing capacity constraint and on the cost of the tests. In particular, contact tracing with group testing is the best alternative conditionally on having enough testing capacity and conditionally on relatively small test costs. In this scenario, and for the benchmark model calibration, the gains are substantial: a reduction of the average mortality of the epidemic by 50%, a reduction of the average output loss by 34%, and a reduction of the average peak prevalence and hospitalizations by, respectively, 58% and 25%. Conversely, In case of a limited testing capacity, or in case of a high test cost, it is best to test the agents only when their symptoms worsen. Random screenings and tests on a voluntary basis appear instead to be sub-optimal alternatives.

The simulations also highlight several additional results. First, the extra-gains of group testing are higher at low testing capacity levels, when lab congestion is more frequent. Second, group testing works even in case of a very limited tracing of the contacts from social activities, which means that it is not necessary to use sophisticated, and privacy-invading, technologies to make it work. Third, the gains of contact tracing with group testing are higher in case of aggressive pathogens (low recovery rates; high probability to develop symptoms; high death probability), although not in case of highly contagious diseases that imply big ratios of positives to tests. Fourth, contact tracing does not yield significant gains in socially dense countries, again because of the big ratios of positives to tests. Fifth, it is not worth allowing voluntary tests when doing contact tracing, because it only exacerbates congestion in testing.

Overall, the paper shows how testing policies influence the evolution of an epidemic through their effect on the observed epidemiological data that, combined with the individual preferences, shape the individual, heterogeneous, responses to the spread of the pathogen.

The idea of using group testing to increase lab capacity is originally from Dorfman [17], who first computed the implied expected reduction in the number of tests needed to screen a population. A huge literature then followed. Aldridge, Johnson and Scarlett [2] survey the most important contributions, while Gollier and Gossner [27] study several applications in the context of the Covid-19 pandemic. My contribution consists in nesting group testing within a SIR-Macro with endogenous responses, and to propose a simple implementation protocol. Jonnerby *et al*. [30], in a related contribution, also propose to pool tests to increase the testing capacity, but requiring a quarantine for all the individuals in the groups that tested positive. My simulation results imply that this is not necessary.

Bergstrom, Bergstrom and Li [8] study the effect of alternative testing strategies on the transmission of a disease, focusing on the relationships between testing intervals, infectiousness and tests characteristics. I complement their analysis by building the testing policies within a SIR model with endogenous responses, and by allowing for group testing. In a related contribution, Galeotti, Steiner and Surico [23] develop a method to evaluate alternative tests on the basis of the policy objective. In my analysis, I simply assume that the tests characteristics are given.

Kasy and Teytelboym [31] propose testing policies based on the likelihood of being infectious, Ely *et al*. [20] study the optimal test allocation problem based on this infection likelihood and Chari, Kirpalani and Phelan [12] compute the implied welfare gains. Brotherhood *et al*. [10] study the effects of age-specific testing policies. Deb, Pai, Vohra and Vohra [15] and Cleevely *et al*. [14] propose instead to target tests based on occupation, wages or geographic attributes. I abstract from tests allocation mechanisms based on personal characteristics in my analysis, although contact tracing is akin to an allocation based on the probability of infection.

More generally, my work is related to the epidemiological literature on SIR models (Kermack and McKendrick [32]; Allen [3]), and to the recent, and fast-developing, literature that merges epidemiological structures into economic models to study the effects of the epidemics, and of the policies to cope with them. Examples of these SIR-Macro models include, among others, Eichenbaum, Rebelo and Trabandt [18], Atkeson [6], Farboodi, Jarosch and Shimer [21], Collard *et al*. [5], Garibaldi, Moen and Pissarides [25], Glover, Heathcote, Krueger and Rios-Rull [26], Krueger, Uhlig and Xie [33] and Jones, Philippon and Venkateswaran [29]. Within this SIR-Macro literature, the closest work are Piguillem and Shi [35], Berger, Herkenhoff, and Mongey [7], Eichenbaum, Rebelo and Trabandt [19] and Pollinger [36], who find that contact tracing policies are more effective than lockdowns at reducing the overall cost of an epidemic. Alvarez, Argente and Lippi [4] come to an opposite conclusion, namely that testing policies cannot be considered as a substitute of a lockdown but, rather, as its complement. A similar argument appears in Dewatripont *et al*. [16]. My contribution to this literature consists in a more detailed, but still tractable, modeling of the testing policies, withing a model where the agents responses are contingent on the observed progression of the epidemic and where the observed progression is itself a function of the implemented testing policy. Bethune and Korinek [9] and Jones *et al*. [29], in two related contributions, highlight the importance of contagion externalities and show that lockdowns are a way to internalize them. Such effects are absent from my analysis.

Caporale *et al*. [11] show that the Covid pandemic had a negative effect on stock returns and on economic activity, and a positive effect on credit default swaps. Akdeniz *et al*. [1], in a related contribution, study the impact of Covid on the oil and gas sector. I study the policies to mitigate the negative effect on activity, but I abstract from the stock market.

## Materials and methods

I build a stochastic Susceptible-Infected-Recovered (SIR) model (Kermack and McKendrick [32]) with production to study the effect of alternative testing policies on the progression of the epidemic and on its consequences. There are five new features that distinguish the model from previous contributions: an optimal choice over getting or not a test; an optimal choice of the extent of social activities; the possibility of developing symptoms independently from the infectious disease; a testing capacity constraint, that limits the number of tests that can be performed each period; a detailed model of the contact tracing technology. Overall, the model features agents that respond to the observed characteristics of the epidemic, and the observed characteristics are themselves a function of the implemented testing policy.

### Agents, firms, schools and diseases

A fictional country without population growth is composed by *N* agents, each represented by a vector *x*_*ij*,*t*_, where *i* indexes the family to which the agent belongs, *j* the school or university she attends or her workplace, and where *t* indexes the time period. At some point, this country is hit by an infectious disease. The vectors *x* is composed of five binary elements that summarize the individual status with respect to the infectious disease. In particular, $x_{ij,t}=\{f_{ij,t},a_{ij,t},s_{ij,t},u_{ij,t},d_{ij,t}\}$, where *f*_*ij*,*t*_ = 1 in case of infection with symptoms, *a*_*ij*,*t*_ = 1 in case of infection without symptoms, *s*_*ij*,*t*_ = 1 in case of susceptibility, to infection, *u*_*ij*,*t*_ = 1 in case of immunity, upon recovery, and *d*_*ij*,*t*_ = 1 in case of death caused by the disease. As in Eichenbaum, Rebelo and Trabandt [18, 19]), I assume that the agents do not know their infection status, which means that tests are valuable. Moreover, I also assume that it is possible to develop the symptoms independently from this infectious disease, so the symptoms are not informative about the infection status, thereby making the tests valuable also for symptomatics. For instance, influenza can induce symptoms which are difficult to distinguish from those induced by Covid-19.

There is a total of *I* families of different sizes, including the possibility of size one to represent singles. Each family is represented as a set *n*_*h*_, with h∈{1,2,…I}. Agents are assigned to families by the function *N*(*i*) = *n*_*h*_ if $i\in n_{h}$. There is also a total of ${\text{J}}^{\text{f}}$ firms and a total of ${\text{J}}^{\text{s}}$ schools, with $J=J^{f}+J^{s}$. Each school/university or firm is represented by a set in $\left\{m_{1},m_{2}\ldots m_{J}\right\}$. The cardinality of each set is either the number of workers employed, if *j* represents a firm, $M_{j}^{f}=|m_{j}|$, $j\in \{1\ldots J^{f}\}$, or the number of students, if *j* is a school or university, $M_{j}^{s}=|m_{j}|$, $j\in \{J^{f}+1\ldots J^{m}\}$. Self-employed individuals are represented as workers in size 1 firms, while I assume that *j* = 0 in case the agent is either unemployed, a pensioner, NEET or if she is simply out of the labor force. For simplicity, I avoid a further division of schools and universities into classes^2^. Workers are randomly assigned to firms and students are randomly assigned to schools, and it is possible, for members of the same family, to be in the same school or firm.

An infection, for a susceptible agent, is the result of a close contact with one of the infected. The infection probability from a matching with a symptomatic is π, while it is $\bar{\pi }<\pi$ in case of a matching with an asymptomatic. In both cases, the probabilities are independent from the history of matches. I also assume that, upon infection, all agents are asymptomatic. The asymptomatics develop symptoms with fixed probability ρ per period. The probability of developing symptoms independently from the infection is instead κ. Symptoms can be mild or severe, both for the infected individuals and for the non-infected that develop symptoms independently. I assume that symptoms worsen with probability β regardless of the underlying infection. I refer to β as the probability of hospitalization, and I assume, for model tractability, that it is always possible for symptoms to worsen independently from the previous history of symptoms severity. Both symptomatics and asymptomatics recover with exogenous probability γ, ignoring that it is, most likely, easier to recover in case of absence of symptoms. Symptomatic agents die with exogenous probability δ. Differently from Favero, Ichino and Rustichini [22] and Eichenbaum, Rebelo and Trabandt [18], among others, I do not model a health system capacity constraint: the death probability does not increase if the number of symptomatics is such that it is impossible to properly treat all of them. As a result, the overall mortality due an uncontrolled epidemic is lower, in the simulation, than its value in case of a health capacity constraint, with the consequence that the gains from mitigation policies that smooth the infection peak are underestimated in my analysis.

### Social participation

All agents engage in social activities, for instance because they use public transportation, because they join a line outside a movie theater, or because they dine with friends at a local restaurant. I model social activities as random matches with agents outside the family, workplace or school, and I assume that they are optimally chosen by the agent depending on progression of the epidemic: agents voluntary decide to change their daily routines to decrease the infection risk to which they are exposed^3^. Absent the epidemic, they would meet with a fixed fraction $\hat{\eta }$ of the population. As the epidemic unfolds, they decrease their social matchings to trade-off the infection risk with the benefits of social activities, for instance an increased consumption of goods and services (restaurant meals, movies etc.) or an increased utility from social interactions. I assume that the perceived contagion risk, at the individual level, grows linearly with the observed disease prevalence. The agents solve the following maximization problem:

$$\max_{\eta_{it}}\;\frac{1}{1-\theta_{i}}\;(N\eta_{it}{)}^{1-\theta_{i}}-\varrho \;(N\eta_{it}){\hat{I}}_{t-1}^{D}\;\;\text{s.t.}\;\eta_{it}\leq \hat{\eta }$$ (1)

where $N\eta_{it}$ is the effective number of matches in period *t* for agent *i* and ${\hat{I}}_{t-1}^{D}=I_{t-1}^{D}/N$ is the observed disease prevalence in *t*–1, equal to the number of discovered infected agents $I_{t-1}^{D}$ divided by the population size. The parameters $\theta_{i}\sim U[0,\bar{\theta }]$ and ϱ govern the shape of the individual responses to the epidemic progression, with low values of $\theta_{i}$ associated with sharper reductions of social matchings, and high values of ϱ associated with reductions that start at smaller values of the observed disease prevalence. For simplicity, I abstract from potential coordination problems, given that everyone could simply free-ride on the social activities reduction of others, with the consequence of an insufficient aggregate reduction^4^. This endogenous response of the agents slows down the epidemic independently from the testing policy, smoothing the peak of infections and the peak number of new hospitalizations, with a resulting reduction of mortality. Moreover, it gives an advantage to the testing policies that are able to discover a bigger number of infected agents, which trigger a stronger endogenous mitigation. For these reason, I discuss, in the on-line appendix, the results from an alternative model simulation with a time-invariant number of social contacts ($\eta_{it}=\hat{\eta }$), showing similar results.

### Testing policies

I build three alternative testing policies in the model. Their goal is to identify and quarantine the infectious, in order to stem the diffusion of the contagious disease. The first consists in allowing voluntary tests only. The second entails random screenings. The third is a contact tracing program, whose goal is to identify all recent matchings of each discovered infected agent. In all cases, I assume that hospitalized agents, with severe symptoms, are always tested, for instance because treatment is contingent on the infection status.

I denote with $\varepsilon_{i,t}\sim Ber(\kappa )$ a binary variable equal to one in case of the onset of symptoms which are not due to infectious disease, and with $\zeta_{i,t}\sim Ber(\beta )$ a binary variable equal to one in case the symptoms worsen, regardless of their underlying cause. Since *f*_*ij*,*t*_ is equal to one in case of an infection with symptoms, I define^5^ with $T_{ij,t}^{h}=\zeta_{i,t}\max\{f_{ij,t},\varepsilon_{ij,t}\}$ the binary variable that describes the hospitalization status of each agent and, as a consequence, the need to take a test to target a medical treatment. In this formulation, I assume that the agents that were infected, and that knew about the infection, still have to take a test upon hospitalization, for instance to check if they effectively recovered or not.

**Contact Tracing**. Contact tracing consists in mandatory tests for those who were matched with a previously discovered infected agent. It is relatively easy to identify the contacts at home, at work or in school, but it is indeed quite complicated to identify all contacts that result from social activities, at least if privacy-invading tracing technologies such as the ones that use cell phone position and security camera images are either not available or not legally allowed. I realistically assume that only a fraction 0≤ϕ≤1 of the contacts from social activities is correctly identified. This fraction ϕ, in turn, is a function both of the efficacy of the allowed tracing technologies and of the laws implemented to stem the epidemic, such as, among others, the introduction of mandatory daily registers of bar and restaurant customers. I use the binary variable *z*_*ij*,*t*_ to indicate if agent *i* is infected and correctly identified as infected in period *t* (see infra for a complete description). I denote with *P*_*ij*,*t*_ the set that includes the individual indexes *i* of all agents that matched with agent *i* as a result of social activities in *t*, and with $P_{ij,t}^{g}\subseteq P_{ij,t}$ with $|P_{ij,t}^{g}|=\phi |P_{ij,t}|$ the set that includes only the indexes of the correctly identified matches, conditional on the tracing efficacy ϕ. Then an agent *i*, is required to take a test, as part of contact tracing, if the binary variable $T_{ij,t}^{g}$ is equal to one, with

$$T_{ij,t}^{g}=(1-z_{ij,t-1})\;1_{\left[i\in (I_{t-1}\cup \;P_{ij,t-1}^{g})\;\vee \;j\in J_{t-1}\right]}$$ (2)

where $I_{t-1}=\{i:z_{ij,t-1}=1\}$ is the set that includes all family identifiers of the discovered infected agents in *t*–1 and where $J_{t-1}=\{j:z_{ij,t-1}=1\}$ is the set that includes all firm and school identifiers of the discovered infected agents in *t*–1. This specification implies that immunes are also tested as part of contact tracing. In fact both the asymptomatics and the symptomatics that recover do not actually know that they are immune if they never took a test while infected. Moreover, those who recovered and who took the test while infected, could be called to prove that they recovered fully with an additional test. Alternatively, the health authorities might be uncertain about the possibility of second infections. The main consequence of this assumption is a worse performance of contact tracing, since testing the previously discovered infected contributes to congestion: if they could simply exhibit a certificate that proves that they were infected, there would be more room to process additional tests each period and a bigger fraction of discovered infected agents. In section [imp], I analyze the performance of contact tracing assuming that the previously discovered infected agents are not tested.

**Voluntary Tests**. A further policy alternative consists in allowing voluntary tests. All agents value the knowledge of their infection status, both for selfish reasons, especially because they can promptly start a treatment, thereby decreasing the probability of hospitalization and death, and for altruistic motives, because they want to reduce the harm that they cause to society as a whole. In both cases, it is reasonable to assume that the benefits of taking the test are an increasing function of the perceived probability to be infected: the higher the probability, the higher the expected harm caused to others and the higher the probability that a medical treatment is needed. I assume that the agents infer the probability to be infected from two observable variables: the presence of symptoms and the observed disease prevalence. I assume that taking the test is costly for an individual, both because of a physical and mental distress, and as a consequence of the out-of-pocket expenditure, and that such costs are idiosyncratic. For simplicity, I also assume that the choice of getting a test and the choice of the number of social contacts are independent, even if the perceived infection risk should be positively associated with social matchings^6^. The agents choose to take a test or not, each period, to maximize the following objective function:

$$U(F({\hat{I}}_{t-1}^{D},\varepsilon_{i,t}))-C_{i}$$ (3)

Where ${\hat{I}}_{t-1}^{D}$ is the observed disease prevalence in *t*–1 and ${\text{C}}^{\text{i}}$ is the individual, time-invariant, disutility/cost of a test. Conditional on the onset of symptoms, there is an individual threshold disease prevalence above which the benefits of tests exceed the cost and, therefore, above which the agent optimally choose to take a test. I denote with ${\hat{q}}_{ij}$ the threshold for asymptomatic individuals and with ${\bar{q}}_{ij}\leq {\hat{q}}_{ij}$ the threshold for symptomatics (it takes a smaller observed disease prevalence to persuade an agent to get tested in case of symptoms). I denote with $T_{ij,t}^{v}$ a binary variable equal to one in case agent *i* voluntarily decides to take a test, with

$$T_{ij,t}^{v}=1_{\left[{\hat{I}}_{t-1}^{D}\geq {\bar{q}}_{ij}\right]}(1-\zeta_{i,t})\max\{f_{ij,t},\varepsilon_{ij,t}\}+1_{\left[{\hat{I}}_{t-1}^{D}\geq {\hat{q}}_{ij}\right]}(s_{ij,t}+a_{ij,t}+u_{ij,t})(1-\varepsilon_{ij,t})$$ (4)

The crucial desirable feature of voluntary testing is that it induces an endogenous mitigation mechanism: the number of tests increases with the observed disease prevalence, which implies a higher number of quarantined infectious that reduces contagions, although the testing capacity constraint might impair this mechanism.

Similarly to contact tracing, I assume that the agents behave as second infection were possible, choosing, in particular, to get tested even if they were discovered as infected in some previous period. This actually implies that a subset of the population of individuals who attach a very high value to the knowledge of the infection status (very small ${\bar{q}}_{ij}$ and ${\hat{q}}_{ij}$) is almost always tested over the course of the epidemic, which is akin to what Eichenbaum, Rebelo and Trabandt [19] assume^7^.

**Random Screenings**. The last policy alternative consists in selecting the individuals to screen at random. I assume that the probability to be selected in this random sample, denoted with ξ, is independent from the previous history of tests. One consequence of this assumption is that it possible, albeit unlikely, to be selected several times for random testing while immune upon recovery. This is the case, for instance, if the authorities are not sure about the possibility of second infections or, alternatively, if the agency that selects the individuals for the random screening does not also access the medical records that keep track of the infection status, or if those records either do not exist or are not updated. The alternative assumption would be to select, for random testing, only the agents that have never been discovered as infected, which might be operationally very difficult to do for a centralized agency, especially at the daily or even weekly frequency, which is the reason why I abstract from this possibility. I denote with $T_{ij,t}^{r}\sim Ber(\xi )$ the binary variable equal to one if the agent is selected for random screenings, which is a draw from a Bernoulli probability distribution with parameter ξ. I assume that such screenings are mandatory, although compliance with such programs could be low, as illustrated by the case of Italy in 2020 (in between the first two Covid-19 waves).

### Lab capacity, rationing, test quality and quarantines

There is a testing capacity constraint in the economy. It can be thought either as of a physical constraint, in the sense that there is not enough equipment and/or professionals to operate them, or as an economic constraint, in the sense that there are not enough resources. In case the total number of tests to process $\Upsilon_{t}$ exceeds the constraint *L*, I assume that there is random rationing. Rationed tests are moved to the next period, and I assume that there is no mandatory quarantine for the agents waiting to take a test of waiting for a result. With limited testing capacity, this would be in fact equivalent to a confinement of potentially non-infectious individuals as if there was a selective lockdown, which will actually be beneficial in terms of mitigation, but which will move the analysis away from its main goal of evaluating alternative testing policies, and not to compare them to lockdowns. Furthermore, it might be also difficult to legally enforce a quarantine without proof of infectiousness, especially because it would only be the byproduct of a health system inefficiency. I also assume that there is no queue: the laboratories do not process leftover tests before others. This assumption is primary motivated by tractability, as it makes it unnecessary to define, and keep track of, a testing priority order. However such an order might exists, for instance because hospitalized patients are typically granted priority, since treatment, and perhaps also their assignment to a hospital, is crucially contingent on the test results.

I define with $\tau_{ij,t}$ the binary variable equal to one in case of a test, either upon hospitalization, as part of contact tracing, as a result of a voluntary choice, as part of random screenings, or because of random rationing in the previous period. It is therefore equal to:

$$\tau_{ij,t}=\alpha_{ij,t}\max\{T_{ij,t}^{v};T_{ij,t}^{g};T_{ij,t}^{l};T_{ij,t}^{r};T_{ij,t}^{h}\}$$ (5)

where $\alpha_{ij,t}$ is the idiosyncratic probability to be tested (non-rationed) in *t*,

$$\alpha_{ij,t}=\left\{ \begin{array}{cc} 1 & \text{if}\;\Upsilon_{t}\leq L\\ \lambda_{ij,t} & \text{if}\;\Upsilon_{t}>L \end{array}\right.$$ (6)

and where $\lambda_{ij,t}\sim Ber(L/\Upsilon_{t})$ is a draw from a Bernoulli distribution with probability equal to the ratio of the maximum number of tests that can be processed in a period *L* (maximum testing capacity) to the total number of tests to perform $\Upsilon_{t}$:

$$\Upsilon_{t}=\overset{N}{\underset{i=1}{\sum }}\max\{T_{ij,t}^{v};T_{ij,t}^{g};T_{ij,t}^{l};T_{ij,t}^{r};T_{ij,t}^{h}\}$$ (7)

For instance, if the agents require, or are required, to take twice as many tests as the system can process, each one of them has only a 50% chance of getting a test. $T_{ij,t}^{l}$ is the binary variable equal to one in case agent *i* was rationed in the previous period:

$$T_{ij,t}^{l}=(1-\alpha_{ij,t-1})\max\{T_{ij,t-1}^{v},T_{ij,t-1}^{g},T_{ij,t-1}^{l},T_{ij,t-1}^{h}\}$$ (8)

I assume that the available tests deliver a positive result, for an infected, with probability σ≤1. Given this sensitivity, an agent *i* is identified as infected in *t* if $z_{ij,t}=\tau_{ij,t}\;S\;(f_{ij,t}+a_{ij,t})=1$, where S~Ber(σ) is a random draw from a Bernoulli distribution with parameter σ. In the on-line appendix, I extend the analysis to tests of lower sensitivity, showing, in line with the results by Larremore *et al*. [34] and Gans [24], that their use, alongside perfect-sensitivity tests, can also mitigate the epidemic. I also assume that the tests have perfect specificity: the test result is always negative if there is no infection. Allowing the use of tests with lower specificity results in unnecessary quarantines, that are desirable for a mitigation perspective, albeit at a high individual cost, but which also makes it more difficult, for the agents, to accept to be tested.

Discovered infected agents are quarantined: they are not allowed to go to work or school and they are not allowed to engage in social activities, although they still keep their relationships with the family members^8^. Actually a quarantine could also entail isolation from the family, say in a hotel room or in a dedicated facility (as in Josè Saramago’s “Blindness"), but I abstract from this, rather extreme, possibility.

Differently from Chari, Kirpalani and Phelan [12], Kasy and Teytelboym [31] and Ely *et al*. [20], among others, I do not explicitly model an optimal allocation mechanism of individuals to tests based on personal characteristics. Depending on the testing regime, the agents are either required to take a test (contact tracing, random screenings) or do it on a voluntary basis. However, the contact tracing strategy, especially when compared to random screenings, can be interpreted as a form of test targeting based on the probability to be infected.

### Group testing

Dorfman [17] was the first to propose group testing to increase the number of processed tests for fixed testing capacity, keeping the same test sensitivity^9^ and specificity. The idea consists in collecting two samples per individual. First samples are bunched into groups of fixed size. If the result for the group is negative, then the result is a negative for all of the group’s members, and second samples are discarded. In case the group result is positive, second samples are processed individually^10^. Suppose that *p* is the probability that a test turns positive and that first samples are bunched in groups of size *g*. The expected number of tests needed for *n* individuals, assuming that the number of groups *n*/*g* is integer and that the test results are independent^11^, is:

$$nG(p,g)=\frac{n}{g}\;\left\{\left(1-p{)}^{g}+(1+g)[1-(1-p{)}^{g}\right]\right\}$$ (9)

Basically with probability $(1-\text{p}{)}^{\text{g}}$, only one test is needed for *g* individuals, while *g* + 1 tests are needed with probability $1-(1-\text{p}{)}^{\text{g}}$, one for thee group and *g* for each member. Minimizing with respect to *g*, for fixed *n* and *p*, gives the optimal group size ${\text{g}}^{*}(\text{p})$ and the resulting number of tests required $\text{n}{\text{G}}^{*}(\text{p})$. The expected testing capacity multiplier upon adoption of the optimal group testing strategy is *S*(*p*) = 1/G^*^(*p*).

In the model, the adoption of group testing makes it possible to overcome the upper bound *L* to the number of tests that can be performed each period. The actual capacity multiplier is a function of the probability *p*, equal to the ratio of positives results to total tests performed, on which the optimal group size depends: the lower this ratio, the lower the probability that group tests will yield a positive and, therefore, the bigger the optimal group size and the lower the number of tests needed to screen the population (bigger capacity multiplier). The problem is that the actual fraction of positives to tests is unknown before the tests are processed, so the labs need to forecast it when setting the group size, and the final capacity multiplier will be a function of how accurate the forecast is. Formally, the actual capacity multiplier in *t*, labeled $S_{t}(p_{t},g(p_{t-1,t}^{e}))$, is a function of the positives to tests ratio *p*_*t*_ and of the optimal group size *g* chosen, according to Dorfman’s solution but based on the forecast of the positives to tests ratio $p_{t-1,t}^{e}$ (forecast of the value in *t* formulated in *t*–1). If the actual ratio of positives to tests is bigger than the forecast, the group size is too big, resulting in more tests needed. Viceversa, if the ratio is smaller than the forecast, less tests are needed.

**Fig 1:**
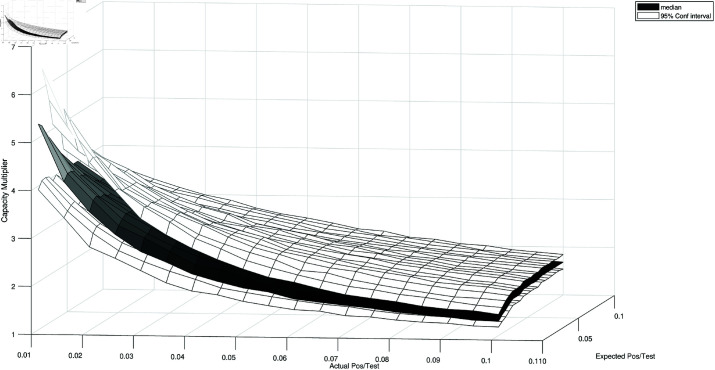
Testing capacity multipliers with group testing. *Notes*: Median and 95% confidence interval of the testing capacity multiplier obtained with group testing in case the actual (ex-post) fraction of positives to tests is different from the forecasted fraction of positives to tests used to set the group size according to Dorfman [17].

The capacity multipliers in case there are differences between the actual and the forecasted ratio of positives to tests can be quantified by simulation. Fig 1 shows the results in case of an actual fraction of positives between 0.1% and 10% and of a group size chosen according to forecasts of the ratio of positives between 0.1% and 10%. I plot the the median capacity multipliers, as well the lower and upper bounds of their 95% confidence interval, over 1000 simulations of societies with 1000 individuals. The pictures shows that gains from group testing could be substantial, although they decrease with the actual fraction of positives.

The evidence summarized in fig 1, together with the results discussed in section [simres], suggests that there could be gains from the introduction of group testing. In the context of an epidemic, those gains could be substantial because of an endogenous reinforcement effect. If there is a binding capacity constraint, only few tests are performed and, with many infections in the economy, this results in big ratios of positives to tests, which make group testing useless. However implementing group testing, by increasing the number of tests, reduces the ratio of positives to tests for fixed number of infected individuals, allowing an even bigger capacity increase that further decreases the positives to tests ratio and so on. In other words, group testing introduces a complementarity that makes it possible to identify and quarantine a big percentage of infectious and, therefore, to reduce infections, hospitalizations and deaths.

A complete group testing strategy entails also a forecast model of the ratio of positives to tests. Actually complicated forecast models are potentially difficult to implement for two reasons. First, and foremost, because there are not enough time series data to estimate or train them, especially at early stages of the epidemic and in case of a new disease such as the Covid-19. Second, the forecasts must be updated very frequently, and individual labs do not necessarily have the know-how to do so, while in case of an external, centralized agency that pools and processes the data, there would coordination and communication problems that are not easy to solve. Given this concerns, I will focus, for simplicity, on a simple adaptive algorithm to set the optimal group size: a rule-of-thumb according to which the forecasts of the positives to test ratio is equal to the positives to test ratio in the previous period: $p_{t-1,t}^{e}=p_{t-1}$. The upper bound to the number of tests that can be performed each period with group testing is therefore:

$$L^{g}=\lfloor L\;S_{t}(p_{t},g(p_{t-1}))\rfloor$$ (10)

For simplicity, I assume that there is a unique lab that sets the group size according to the previous period positives to tests ratio. The tests are randomly bunched in groups and processed according to the group testing protocol. I then compute the total number of tests processed each period and the total number of tested agents each period.

### Production

The production side of the economy is stylized, since the firms are not active decision makers in the testing policy: their only role is to determine production given the number of available workers. I assume that there is no capital, and that the technology does not change over time, so production depends only on employment. I assume that the infected agents are quarantined only in case they tested positive, in which case they cannot work. All others, including the infected with symptoms and the symptomatic, but non-infected, go to work, unless in case of severe symptoms that require hospitalization. I assume that the firms cannot temporarily replace the quarantined workers with the unemployed and, since there is no population growth, it is also impossible for them to hire new workers in case of deaths. The number of workers employed a firm *j*, at any point in time, is therefore equal to:

$${\hat{m}}_{j,t}=|m_{j}|-\sum_{j\in m_{j}}\{d_{ij,t}+\max[(1-Z_{ij,t})(f_{ij,t}+a_{ij,t})\;,\;T_{ij,t}^{h}]\}$$ (11)

where *Z*_*ij*,*t*_ is equal to one if the agent *i* was never discovered as infected before period *t*, with $Z_{ij,t}=\prod_{\bar{t}=1}^{t}(1-z_{ij,\bar{t}})$. Production, for each firm, is equal to $y_{j,t}={\hat{m}}_{j,t}^{\alpha }$, while aggregate production is simply obtained summing the individual firms’ output: $Y_{t}=\sum_{j=1}^{J^{f}}y_{j,t}$. For simplicity, I will assume, throughout the analysis, that α=1. The output gap, in this economy, is the percentage difference between aggregate production and potential production, the latter defined as what would be produced without the epidemic and without any implemented policy, so without deaths, quarantines and hospitalizations: ${\bar{Y}}_{t}=\sum_{j=1}^{J^{f}}|m_{j}{|}^{\alpha }$. It follows that the testing policies have two contrasting effects on the output gap: the bigger is the number of discovered infected agents, the bigger is the output reduction due to the quarantines; more discovered infected, however, reduce infections and the overall mortality rate, with a lower long-run output reduction.

### Dynamics

The model features five dynamic equations for changes of status with respect to the disease. An agent is infected and symptomatic, in a given period, if she was infected and symptomatic in the previous period and if she did not recover or die, or if it was asymptomatic in the previous period and developed symptoms:

$$f_{ij,t+1}=(1-\Delta )(1-\Gamma )f_{ij,t}+P(1-\Gamma )a_{ij,t}$$ (12)

where Δ~Ber(δ), Γ~Ber(γ) and P~Ber(ρ) is a Bernoulli random variable with probability equal to the probability of developing symptoms ρ. An agent is asymptomatic if it was asymptomatic in the previous period and did not recover and did not develop symptoms, or if it was susceptible and infected:

$$a_{ij,t+1}=(1-\Gamma )(1-P)a_{ij,t}+H_{ij,t}s_{ij,t}$$ (13)

where the binary variable $H_{ij\;t}$ equal to one in case of infection:

$$H_{\;ij,t}=\left\{ \begin{array}{cc} 1 & \text{prob}\;1-{\left(1-\pi \right)}^{{\bar{F}}_{i,t}}{\left(1-\bar{\pi }\right)}^{{\bar{A}}_{ij,t}}\\ 0 & \text{otherwise} \end{array}\right.$$ (14)

${\bar{F}}_{i,t}$ is the number of symptomatics with whom each susceptible is matched:

$${\bar{F}}_{i,t}=\;\sum_{\left\{\tilde{i}\in N(i)\;;\;\tilde{i}\neq i\right\}}\;f_{\;\tilde{i}j,t}\;+\;\;\sum_{\left\{\tilde{j}\in m_{j}\;;\;\tilde{j}\neq j\right\}}\;f_{\;i\tilde{j},t}Z_{\;i\tilde{j},t}\;+\;\eta_{ij,t}^{f}\;\sum_{\left\{\tilde{i}\notin N(i)\;;\;\tilde{j}\notin m_{j}\right\}}\;f_{\;\tilde{i}\tilde{j},t}Z_{\;\tilde{i}\tilde{j},t}$$ (15)

where the first term is the number of symptomatics in her family, the second is the number of never-discovered symptomatics in her school or workplace and the third is a fraction $\eta_{ij,t}^{f}$ of the never-discovered symptomatics met as part of social activities. The number of asymptomatics with whom each susceptible is matched is instead:

$${\bar{A}}_{i,t}=\;\sum_{\left\{\tilde{i}\in N(i)\;;\;\tilde{i}\neq i\right\}}\;a_{\;\tilde{i}j,t}\;+\;\;\sum_{\left\{\tilde{j}\in m_{j}\;;\;\tilde{j}\neq j\right\}}\;\;a_{\;i\tilde{j},t}Z_{i\tilde{j},t}\;+\;\eta_{ij,t}^{a}\;\sum_{\left\{\tilde{j}\notin m_{j}\;;\;\tilde{i}\notin N(i)\right\}}\;\;a_{\tilde{i}\tilde{j},t}Z_{\tilde{i}\tilde{j},t}$$ (16)

where, again, the first term is for asymptomatics in the family, the second for never-discovered asymptomatics on the workplace or in school, and third is a fraction $\eta_{ij,t}^{a}$ of the never-discovered asymptomatics met in social activities. Since I abstract from the possibility of second infection, I have that an agent is immune if it was immune in the previous period or if she recovered from an infection:

$$u_{\;ij,t+1}=\Gamma f_{\;ij,t}+\Gamma a_{\;ij,t}+u_{\;ij,t}$$ (17)

An agent is instead susceptible if it was susceptible in the previous period and if she was not infected:

$$s_{\;ij,t+1}=s_{\;ij,t}(1-H_{ij\;t})$$ (18)

Finally, infection can result in a death:

$$d_{\;ij,t+1}=\Delta (1-\Gamma )f_{\;ij,t}+d_{\;ij,t}$$ (19)

### The government

I assume that the government optimally chooses the testing policy *W* in order to minimize the following loss function:

$$\min_{W}\;\omega \;[\sum_{i=1}^{I}mvl\cdot d_{ij,T}^{W}]+(1-\omega )[\sum_{t=1T}(Y_{t}^{W}-{\bar{Y}}_{t})+\sum_{t=1T}c\Upsilon_{t}^{W}]+O(W)$$ (20)

where the superscript indicate the implemented testing policy. The first term is the total number of deaths at the end of the epidemic (period T) multiplied by a monetized value of life *mvl*. The second term is the sum of the cumulative output loss, equal to the difference between actual and potential output, and of the total cost of the tests, equal to the product of the fixed test cost *c* times the number of tests performed over the course of the epidemic. For simplicity, I assume, in case of group testing, that the number of tests performed is equal to the number of processed swabs, as if the cost of the non-processed swabs was zero. The third term *O*(*W*) is the organizational cost of the testing strategy. Most likely, contact tracing policies are more costly to implement than voluntary tests and random screenings because they are technology intense and because they require specialized professionals. The weight ω reflects the relative importance assigned to deaths vis-a-vis the other (pure) monetary losses associated with the epidemic. I consider three alternative scenarios: ω=1, with a government concerned only with deaths (and where *mvl* is not influential); ω=0, with deaths that are not valued by the government; ω=0.5, in which case the government is concerned about deaths only to the extent to which they determine an output loss.

The policy options in the set *W* are 7: testing only the hospitalized; allowing tests on a voluntary basis, with or without group testing; implementing random screenings, with or without group testing; contact tracing, with or without group testing. In case of group testing, the group size is optimally set according to the observed positives to tests ratio in the previous period (adaptive model). In case of random screenings, I assume that thee government optimally chooses the value of ξ that minimizes the expected value of the loss function. I also assume that the tests to the hospitalized are mandatory under all testing policy alternatives. Other than that, I do not allow for mixing among the available testing policies for simplicity. This formulation implicitly assumes that the government directly manages the labs and that there is no out-of-pocket expense for the agents that get tested. In case of voluntary tests, this implies that the government effectively subsidized the individual choice of testing, the rationale being that there are positive externalities to screenings. This subsidy could be higher than optimal, but I abstract from this optimal subsidization problem for simplicity.

### Calibration

I calibrate the model to Italy using the available information on Covid-19. Table 1 summarizes the model parameters used in the baseline simulation^12^.

**Table 1:** Calibration.

Parameter	Description	Value	Target
π	Infection probability, symptomatics	0.02	Covid-19 R (Chowdhury *et al*. [13])
$\bar{\pi }$	Infection probability, asymptomatics	0.01	Covid-19 R (Chowdhury *et al*. [13])
ρ	Probability of symptoms if infected	0.25	Covid-19 stats (ISS)
κ	Probability of symptoms if not infected	0.07	Influenza prevalence (ISS)
β	Hospitalization probability if symptoms	0.05	Brotherhood *et al*. [10]
γ	Recovery probability	0.1	Covid-19 stats (ISS)
δ	Death probability	0.005	Covid-19 death rate (ISS)
σ	Test sensitivity	1	RT-PCR tests for Covid-19
*I*	Number of Families	500	Simulation scale
$\bar{n}$	Average family size	2.5	Census data
$\bar{\eta }$	Pre-epidemic density of the economy	0.005	Social participation data (ISS)
*W*	Share of workers	0.6	Labor force participation
${\text{J}}^{\text{max}}$	Max firm size	12	Average firm size; Numb of small firms
${\text{J}}^{\text{f}}$	Number of firms	64	Average firm size; Numb of small firms
${\text{J}}^{\text{s}}$	Numb of schools	5	Average school size
*mvl*	Monetized value of life	20	Alvarez *et al*. [4]
$\bar{\theta }$	Density reduction, upper bound	10	Heterogeneous individual responses
ϕ	Percentage of traced social contacts	0.5	Assumption
*Q*	Preference for voluntary tests	0.52	Assumption

I simulate an economy with *I* = 500 families, whose size is set according to the Italian Institute of Statistics (ISTAT) data for 2019: one member for 31% of the families, two members for 27% of them, three for 20%, four for 16% and five or more for 6%. Given the relatively small simulation, I exclude the possibility of more than 5 members. The resulting number of agents *N* is close to 1200, and the average family size $\bar{n}=N/I\approx 2.5$.

I set the labor force participation such that *W* = *L*/*N* = 0.6, consistently with ISTAT. Since, according to ISTAT, 44% of the workers are employed in small firms, I set the number of firms with one employee so that they account for 44% of the stock of all firms. I calibrate the maximum firm size ${\text{J}}^{\text{max}}$ and the number of firms ${\text{J}}^{\text{f}}$, to match the ISTAT average of 3.87 employees per firm, and the 56% fraction of workers employed in non-small firms (of size bigger than one in the simulated economy). The resulting values are *J*^*max*^ = 12 and *J*^*f*^ = 64. I assume that only the agents living in families of three or more can attend schools or university, thereby abstracting from the possibility of single parents. To match ISTAT data, I fix the fraction of those agents to 38.5%. I then calibrate the number of schools ${\text{J}}^{\text{s}}$ so that each school, of equal size, accounts for 2.2% of all school age pupils, once again consistently with ISTAT data. Allowing for schools of different size will only introduce more noise in the simulation results, since the diffusion of the disease will also depend on school size (higher size implies a faster diffusion), but I abstract from this feature.

I calibrate the number of random matches from social activities in the absence of an epidemic (or in case of a zero observed prevalence) $\hat{\eta }$ in order to have an average of 18.5 total matches per individual, consistently with the estimates by the Istituto Superiore di Sanità (ISS) [28]. These total number of matches *TM*_*ij*_ for an individual *i*, living in family *n*_*i*_ and going to work or school to *m*_*j*_ is equal to $TM_{ij}=|n_{i}|\;-\;1\;+\;|m_{j}|\;-\;1_{\left[|m_{j}|>0\right]}\;+\;\hat{\eta }N$, where the indicator function in the middle term accounts for the possibility of agents not working and not going to school (NEET, pensioners, housewives etc.). Solving $(1/N)\sum_{i=1}^{N}TM_{ij}=18.5$, I obtain, for the simulated population size, $\hat{\eta }=0.005$.

Ideally the individual adjustment of social contacts in response to the epidemic should be calibrated looking at the individual responses to the covid-19 outbreak in 2019. The problem is that most countries, including Italy, implemented lockdown mitigation policies to reduce social contacts independently from the individuals’ will. In the absence of more detailed information, I set the upper bound $\bar{\theta }$ to 10, in order to allow for a wide range of individual responses^13^. I assume that $\varrho =\varrho_{i}$ is idiosyncratic and such that all agents start reacting to the epidemic as soon as the observed disease prevalence is above 0.5% (below this threshold, they all simply choose $\eta_{it}=\hat{\eta }$). Operationally, I set $0.005\cdot \varrho_{i}=(\hat{\eta_{i}}N{)}^{-\theta_{i}}$, according to the first order condition of the utility maximization problem over $\eta_{i}$ with ${\hat{I}}_{t-1}^{D}=0.005$. This threshold choice is actually arbitrary, but the results appeared to be robust in a neighborhood of 0.5%. Higher values are actually unlikely to be reasonable, as they substantially imply the absence of an endogenous response to the epidemic, which is a case I discuss in appendix. Notice that if the endogenous response to the epidemic is strong, the testing policies are not necessary. Thus a generalized voluntary lockdown at low prevalence rates makes the whole numerical exercise uninteresting.

Given the value of $\eta_{it}$, I draw randomly, each period, the number of symptomatics, asymptomatics, susceptibles, and immunes with whom each agent is matched in social activities. In particular, I extract, for each agent, a random subset of $\eta_{it}N$ elements from the subset *O*_*ij*,*t*_ of the population of individuals who are not in the agent *i* family, workplace or school and who are not infected and discovered as such in period *t*. Then I count the number of symptomatics in this set and, dividing it by the total number of symptomatics in *O*_*ij*,*t*_, I obtain the share $\eta_{ij,t}^{f}$. I proceed similarly for asymptomatics, immunes and susceptibles.

In order to make the model more realistic, I assume, in line with Eichenbaum, Rebelo and Trabandt [18], that a subset of the population, with a higher individual health risk, has twice as much chances of dying of Covid-19, and I set this this fraction to 17% in line with the percentage of the population above 70 in Italy. Then I set the per-period death probability δ in order to match the observed total mortality of Covid-19 as of March 2021, equal to 3.1%. The resulting calibrated value is δ=0.5%. Given the calibrated value of δ, the value of the the probability to recover that delivers the observed level of recovered/immunes (infected-deaths) in the same period is γ=0.1.

I set the probability to develop symptoms in case of infection to ρ=0.25 in line with ISS data (average in 2021). Without an infection, and independently from it, there is a probability κ=0.07 to develop similar symptoms to the infectious diseases, where 7% is the influenza prevalence registered in Italy in 2019 by the ISS. Thus, in the Covid-19 example, the baseline model simulation refers to the Influenza season, but I will also consider an alternative, summer-like, scenario with a very small incidence of symptoms without infection (see section).In line with Brotherhood *et al*. [10], I assume that hospitalization are required, on average, for 5% of the agents with symptoms, but independently from the infection status: β=0.05. I set the transmission probability from an asymptomatic to $\bar{\pi }=1\%$ and I assume that it is twice more likely to get the infection from a symptomatic than from an asymptomatic, so π=2%. Assuming that the period, in the model, is equivalent to one day, the implied reproduction number is R=18.5·7·0.0125=1.618 per week, in line with the estimates by Chowdhury *et al*. [13] for Covid-19.

For simplicity, I assume that the agents utility function in case of voluntary tests is simply equal to the perceived probability to be infected, $U(F({\hat{I}}_{t-1}^{D},\varepsilon_{i,t}))=F({\hat{I}}_{t-1}^{D},\varepsilon_{i,t})$. The perceived probability, in turn, is a linear function in both arguments: $F({\hat{I}}_{t-1}^{D},\varepsilon_{i,t})=\Upsilon^{I}{\hat{I}}_{t-1}^{D}\;+\;\Upsilon_{i}^{E}\varepsilon_{i,t}$, with the peculiarity that the dependence on the onset of symptoms is agent specific, potentially being a function of the individual health status, while the dependency on the epidemic evolution is constant across individuals. I assume that the idiosyncratic disutilities *C*_*i*_ are uniformly distributed: $C_{i}\sim U[0,\bar{C}]$. This implies that the individual thresholds for asymptomatics will also be uniformly distributed: ${\hat{q}}_{ij}\sim U[0,Q(\Upsilon^{I},\bar{C})]$. *Q* is the observed disease prevalence big enough to persuade all asymptomatics to take a test, and it is a function of the dependency of the perceived probability on the disease prevalence $\Upsilon^{I}$ and of the upper bound for the individual costs $\bar{C}$. As for the coefficients $\Upsilon_{i}^{E}$, I draw them randomly such that ${\bar{q}}_{ij}\sim U[0,{\hat{q}}_{ij}]$ which implies a smaller disease prevalence above which a test maximizes the individual utility in case of symptoms. I also assume that there is no extra disutility for the agents that take two swabs in case of group testing (sometimes swabs are repeatedly inserted even in case just one sample is needed). Unfortunately there is not much information available on the preferences over tests. I calibrate the upper bound *Q* to the uniform distribution of threshold values for the asymptomatics so that 50% of the symptomatics (recall that the threshold value for a symptomatic is a draw from a uniform distribution between 0 and the threshold value in case she develops symptoms) voluntary decide to take a test when the observed disease prevalence is equal to 10%. The resulting value is *Q* = 0.52. The smaller is *Q*, the more effective is voluntary testing at fighting the epidemic because of the bigger number of individuals who choose to get a test for given observed prevalence. Since I set this parameter rather arbitrarily, I analyze the robustness of the results in section [rob], allowing, in particular, for a much smaller *Q*.

The tests available have perfect sensitivity, σ=1, which is nearly true for RT-PCR (Real Time Polymerase Chain Reaction) tests on naso-pharyngeal swabs in the case of Covid-19, at least for non-negligible viral loads. I study the effect of less sensitive tests in appendix. I assume a 50% efficacy of the tracing technology in the baseline simulation, so ϕ=0.5, which means that only half of the random matches from social activities are correctly identified for each individual. I study extensively the effects of policies that entail a change of this fraction in section [imp]. The average fraction of the population tested each period in case of random screenings ξ is optimally chosen by the Government. Notice that it is not optimal to fully exhaust testing capacity with random screenings to leave room to promptly process tests in hospitals, which might be substantial near the peak of the epidemic. Notice also that, in reality, in order to reach the desired level of ξ, the government must ask to a bigger fraction of agents than ξ to get tested, as compliance with such screening programs programs is typically low, while the enforcement of mandatory screenings that could be even more complicated than the enforcement of a lockdown. Contact tracing, conversely, is much easier to enforce because the traced agents have a higher risk of being infected, typically resulting in a bigger compliance.

I set the monetized value of life *mvl* used by the government to evaluate the cost of deaths to 20 times the GDP per capita over the course of the epidemic (total potential production between period 1 and period *T* divided by total population), in line with Alvarez, Argente and Lippi [4]. The cost of each test *c* is set as a fraction of daily GDP per capita, which in Italy is around 75 euros. In particular, I will compute optimal testing policies for different values between 1% (0.75 euros) and 100% of daily GDP per capita.

Note that it is very difficult to precisely calibrate the model to real world epidemiological data on Covid-19, mostly because the virus mutates frequently (for instance the more contagious“Delta" strain became prevalent in Italy during the summer of 2020, to then be replace by the much more contagious but less deadly “Omicron" at the end of 2021), which means that the exact prevalence of each strain is difficult to track, and because the testing policies, which vary at the local level, changed a lot over time and were not publicly disclosed^14^ (priority order in labs, two versus one negative swab to be legally considered recovered, authorizations to labs to perform tests, ban on voluntary tests etc.). Thus the scope of the calibration exercise is not to match the data but, rather, to make the model operational for policy analysis. In section [rob], I analyze the robustness of the simulation results to alternative parameters, which also addresses the external validity of the results.

I simulate 500 epidemics conditional on the testing policies. The epidemics start with the random infection of four agents. The testing policies start^15^ ten periods after the first infections. I discard the simulations without an epidemic, which is a rather unlikely, albeit possible, event, that can be observed in case the initially infected individuals recover before spreading the disease. In subsection ?? I discuss the optimal policy. In subsection [sim_res] I discuss instead the results obtained with the best policy alternative, contact tracing, in greater detail. In the On-line appendix, I further discuss the results obtained with voluntary tests and random screenings.

Overall, the epidemic dynamics under different testing policies are very heterogeneous, and so are its observed characteristics. With voluntary tests, these differences are actually also a by-product of different individual preferences. This actually means that, when comparing the evolution of the epidemic in different countries or areas, it is important to account for the implemented testing policies.

## Results and discussion

The optimal policy is contingent on four parameters (given that ξ is chosen optimally): the weight given to deaths in the objective function ω, the individual cost of a test *c*, the testing capacity constraint and the extra organizational cost of the testing strategy with respect to the no-policy alternative *O*(*W*). For given testing capacity constraint and for given weight ω, I look for the optimal policy as a function of the test cost *c* assuming that O(W)=0∀W, and then compute the values of the organizational cost at which the optimal policy changes.

**Fig 2:**
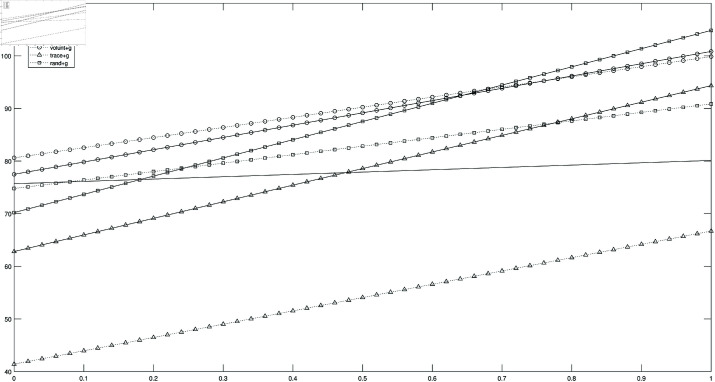
Loss functions, ω=0.5. *Notes*: Loss functions (see equation 20) in units of total GDP per capita as a function of the test cost. Testing capacity equal to 5% and weight to the monetized value of deaths ω equal to 0.5. Solid lines: no group testing. Dotted lines: group testing. Triangles: contact tracing. Squares: random screenings. Circles: voluntary tests. The parameters used for the simulation are summarized in table 1. Optimal random screening strategy entails ξ=0.9.

I first consider the case of an equal weight placed by the policy maker to deaths and output, ω=0.5. Fig 2 plots the loss functions of the 7 testing strategies as a function of the test cost *c* in case of a 5% testing capacity. All loss functions are in units of total GDP per capita (total production over the duration of the epidemic divided by pre-epidemic population size). Absent organizational costs, the first-best consists of contact tracing with group testing regardless of the value of *c*. The second best policy is instead a function of the test cost: contact tracing without group testing when *c* is below 50% of daily GDP per capita, and tests only to the hospitalized for higher costs. For high values of *c*, the extra gains of contact tracing in terms of reduced mortality are washed out by the increased costs of testing, and it is worth implementing only in conjunction with group testing. In general, the higher is the test cost, the smaller are the gains from contact tracing. Voluntary tests, on the other hand, are always dominated choices: they do not allow the identification of a sufficiently big share of the infected to slow down infections and they involve a cost. Random screenings with the optimal choice of ξ yield instead intermediate results. As for the organizational costs, fig 2 shows that, at small values of *c*, group testing in addition to contact tracing is worth implementing only if its organizational cost is smaller than about 20 times the total GDP per capita, which is likely to be the case since the extra organizational cost of group testing is small. If however contact tracing with group testing costs more than 30 times the total GDP per capita, it is best to opt for random screenings. The situation is different at high values of *c*: contact tracing alone does not improve upon the performance of the no-policy alternative and even a small organizational cost of contact tracing with group testing is sufficient to make it not worthwhile.

For the benchmark *mvl* equal to 20 times the GDP per capita, the effective weight assigned to deaths in the loss function is quite high, so that the optimal policy in case of ω=1 is the same as the optimal policy in case of ω=0.5. For ω=0, and in the baseline 5% testing capacity benchmark, the gains from contact tracing are, in general, smaller, as their main effect, the reduction of deaths, is not valued anymore by the policy-maker. In this case, it takes a smaller organizational cost for the no-policy alternative to be preferred to contact tracing with group testing, even at low levels of the test cost *c*. For instance, if this cost exceeds 20% of daily GDP per capita, the no-policy alternative is actually the best policy.

In case of a bigger testing capacity equal to 10%, the gains from contact tracing are higher and, absent any organizational cost, contact tracing with group testing is still the first best policy regardless of the test cost. However, at low levels of *c*, the extra gains from group testing are actually very small, because the labs can already process all the required test required to identify a high fraction of the infected, and the extra capacity created by group testing is only a minor cost-saving device, without any gain in terms of deaths. With an organizational cost, and for high levels of the test cost *c*, which is the worse-case scenario for contact tracing, contact tracing with group testing is worth implementing it its cost is less than 40 times the total GDP per capita, otherwise it is best to test only the hospitalized.

In case of a very small testing capacity of 1%, contact tracing does not work because of congestion in the labs: the number of required tests grows very rapidly with the infections at early stages of the epidemic and the labs are not able to promptly process all of them^16^ and, since the positives to test ratio is also high in case of a small testing capacity, group testing is not helpful. Random screenings are also limited in scope because of the small test capacity, and group testing, again, does not help because of the high positives-to-test ratio. Voluntary tests, on the other hand, impose a cost without being able to identify a sufficiently big number of the infected to slow down the epidemic. At such low capacity levels, the best policy consists in testing only the hospitalized.

Summarizing, the gains from contact tracing are higher in case of a high testing capacity and in case of a small test cost, while the extra gains from group testing are smaller at high testing capacities. At small capacity levels, or at intermediate capacity levels but with high test costs, it is best to test only the hospitalized.

### Testing policies performances

Table 2 compares, for a 5% maximum test capacity, the performance of the three best testing policies: contact tracing with group testing (and rule-of-thumb forecasts), contact tracing without group testing and the no-testing policy benchmark (tests only to the hospitalized).

**Table 2:** Contact Tracing and Group Testing.

	Test	Mean	Median	Std	95% Conf	
Infpeak	Hos	16.89	16.68	1.97	13.74	19.82
	Trace	11.55	12.33	4.62	0.58	17.10
	Trace+Group	7.01	7.25	3.75	1.14	13.21
Hospeak	Hos	1.39	1.38	0.22	1.14	1.71
	Trace	1.27	1.22	0.23	0.89	1.55
	Trace+Group	1.04	1.06	0.14	0.81	1.22
Deaths	Hos	2.58	2.53	0.44	1.95	3.18
	Trace	2.05	2.11	0.88	0	2.71
	Trace+Group	1.27	1.31	0.75	0	2.36
Yloss	Hos	-2.75	-2.76	0.46	-3.49	-2.04
	Trce	-2.48	-2.65	0.92	-3.47	-0.16
	Trace+Group	-1.82	-1.88	1.04	-3.45	-0.18
Tests	Hos	0.71	0.71	0.02	0.66	0.74
	Trace	5.07	5.39	1.48	0.98	6.45
	Trace+Group	4.08	4.40	1.34	1.10	5.58
Screened	Hos	0.71	0.71	0.02	0.66	0.74
	Trace	5.07	5.39	1.48	0.98	6.45
	Trace+Group	7.86	8.35	2.51	1.96	10.48
Discovered	Hos	27.14	26.76	3.01	23.24	32.97
	Trace	44.91	43.73	4.26	39.17	50.01
	Trace+Group	61.78	59.11	10.03	49.27	81.38
Postest	Hos	46.44	45.80	3.46	40.83	50.01
	Trace	5.72	6.31	1.45	3.28	8.19
	Trace+Group	7.71	7.91	1.10	5.72	8.77

**Notes**: Infpeak is the percentage of the population infected at the peak of the epidemic (peak actual prevalence). Hospeak if the maximum fraction of the population to access an hospital. Deaths is the mortality rate as a percentage of the population, computed at the end of the epidemic. Yloss is the cumulative output loss (total output gap) gross of the test cost and of the organizational cost of the testing policy. Tests is the total number of tests performed over the course of the epidemic expressed in multiples of the population. Discovered is median of the ratio of discovered infected to total infected during the epidemic. Postest is the median ratio of new discovered infections to total performed tests during the epidemic. Screened is the effective number of tests ()bigger than the number of tests performed because of group testing. Test refers to the testing policy. Hos refers to tests only to the hospitalized agents. Trace refers to contact tracing and tests to hospitalized agents. Trace+Group refers to group testing in two rounds (1 grouping) in addition to contact tracing and tests to hospitalized agents only. Mean, median, standard deviation (Std), and 95% confidence interval (95% Conf) refer to the distribution of the results over all simulation runs with an epidemic. The parameters used in the simulation are summarized in table 1.

The main advantage of group testing is the higher number of screened individuals for the same testing capacity: the median percentage of screened individuals increases by 55% with respect to contact tracing alone. The result is an increase in the median percentage of identified infected up to 62%, or 37% more with respect to contact tracing alone and 2.3 times as big as in the benchmark with tests to the hospitalized only. Having more quarantined infectious, on the other hand, smooths the peak of the epidemic, with an average peak infection prevalence that drops, from the no-policy benchmark, by 58%, and with an average peak in new hospitalizations that drops by 25%. Average mortality, as a consequence, drops, and by a significant amount: 38% with respect to contact tracing alone, and 51% with respect to the alternative of testing only the hospitalized. A lower average mortality under contact tracing with group testing, in turn, reduces the long-run output loss, but the bigger number of quarantined infectious increases the short-run output loss. Overall, the effect is a smaller output loss: by 26% than under the alternative contact tracing, and by 34% than benchmark. In addition, the average total number of tests performed with group testing and contact tracing decreases by 20% with respect to contact tracing, so group testing also eases the pressure on labs and reduces the costs of testing.

**Fig 3:**
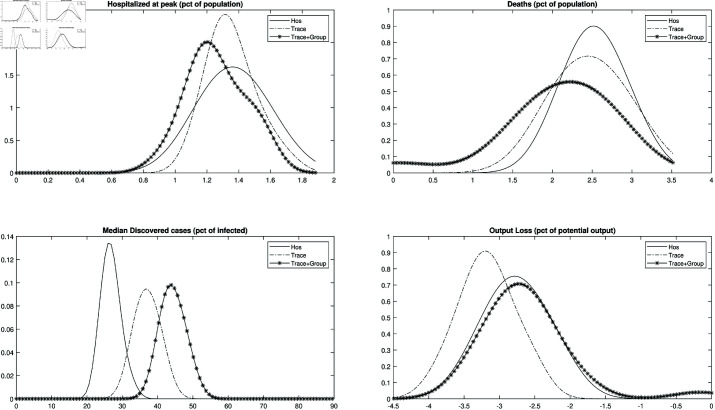
Contact tracing and group testing during an epidemic. *Notes*: Empirical distributions of the peak in new hospitalizations (upper-left panel), mortality (upper-right panel), median percentage of discovered infected (lower-left panel) and output gap (lower-right panel) over simulation runs. Solid line: tests to hospitalized agents only; Solid-dashed line: contact tracing; Solid line with asterisks: contact tracing with group testing. The parameters used for the simulation are summarized in table 1.

Fig 3 shows the empirical distributions (over simulation runs) of the mortality rate, of the peak hospitalization rate, of the median percentage of discovered infected and of the total output loss, for the baseline model simulation. With respect to the no-policy benchmark, the distributions of both hospitalizations and deaths shift to he left in case of contact tracing, and the more so in case of group testing, while the distribution of discovered cases shifts to the right. Total output loss with contact tracing and group testing is instead close to the no-policy benchmark, although there is a higher probability mass on very small losses.

All in all, the gains that can be achieved with contact tracing and group testing, at the benchmark testing capacity, are substantial.

### Improving upon contact tracing and group testing

In this section I study how to improve contact tracing along three dimensions: adopting more effective tracing technologies, allowing individuals to voluntary take tests even if they are not required to do so, and avoiding tests to the previously discovered as infected. As for group testing, I consider an alternative implementation with the collection of three samples per individual and two rounds of grouping.

**Limited tracing capacity**. The choice of the efficacy at tracing social contacts ϕ=0.5 in the baseline simulation is rather arbitrary. As already stressed, this parameter is a function of both the tracing technologies, which range from customers registries filled by restaurants and shops to cell phone localization and security cameras image recognition, and of the legal framework which might or might not allow intrusive and privacy-violating practices without explicit consent. Since there might be significant cross country differences both in the ability to manage those technologies and in their legal feasibility, it is interesting to assess the performance of contact tracing for different tracing efficacy. I simulated the model with two alternative values of ϕ, respectively 25%, meaning that only one out of four contacts from social activities is correctly identified and tested, and 75%. The results turned out to be similar to the baseline simulations with ϕ=0.5. This implies that contact tracing might collapse because of a binding test capacity constraint and not because of an insufficient number of identified social contacts. Putting it differently, sophisticated tracing technologies are not very useful if there is not enough testing capacity: for small capacity, tracing within the family, school and workplace is enough to create congestion, and the extra gains from a correct identification of a bigger percentage of the social contacts are minimal. In terms of the loss function, a lower tracing efficacy implies a smaller number of tests performed which, for a similar death rate, delivers a smaller loss function.

In the limit, when it is impossible to trace social contacts (ϕ=0), the gains from contact tracing are modest. Nevertheless, for the baseline 5% testing capacity and in the absence of organizational costs, contact tracing with group testing is still the first best policy, unless the cost *c* is bigger than 90% of daily GDP per capita, in which case the no-policy benchmark is better. The organizational cost that makes it worthwhile to implement contact tracing with group testing is, in general, smaller in this limited tracing scenario. For instance, for *c* = 0.7, it takes a cost of about 7 times the total GDP per capita to prefer the no-policy alternative over contact tracing with group testing. At the 10% capacity, and in the absence of organizational costs, contact tracing with group testing is better than the no-policy alternative regardless of the test cost and even for relatively high organizational costs, as in the benchmark simulation with a 50% tracing efficacy. The only difference in this alternative scenario is that the loss function with random screenings and group testing is very close to the loss function of contact tracing and group testing.

In conclusion, when deciding whether to implement contact tracing, it is best to focus the efforts on increasing the testing capacity, rather than adopting sophisticated, and potentially privacy-invading, tracing technologies. Group testing helps, but not if the testing capacity is very small.

**Additional voluntary tests**. I simulated the model allowing voluntary tests in addition to contact tracing. The idea is that, since symptomatic agents take the test frequently, this can allow the identification of more of the infectious, therefore improving upon the performance of contact tracing alone. The problem, however, is that the capacity constraint becomes binding more often, and, since the extra tested agents have a lower probability of being infected, the resulting congestion might reduce the overall percentage of identified infected. The simulation results show that the two effects balance, and the overall performance of contact tracing is almost unchanged, even in case of a 10% maximum capacity. The conclusion is that allowing voluntary tests, when doing contact tracing, is not worthwhile.

**Immunes are not tested**. One of the strong assumptions of my simulation exercises is that all testing policies, including contact tracing, are designed as if second infections were possible. In particular, agents are required to take tests, as part of contact tracing, even if they took a test while infected in some previous period, so even if they are immune and if they know about their immunity. The resulting congestion in the labs dwarfs the performance of contact tracing. To evaluate the importance of this assumption, I simulated the model again assuming that identified infected agents are not tested when contact tracing is implemented. Thus an agent is required to take a test, under contact tracing, if the binary variable ${\bar{T}}_{ij,t}^{g}$ is equal to one, with:

$${\bar{T}}_{ij,t}^{g}=Z_{ij,t-1}\;1_{\left[i\in (I_{t-1}\cup \;P_{ij,t-1}^{g})\;\text{or}\;j\in J_{t-1}\right]}$$ (21)

For the baseline model parameters, the testing capacity constraint becomes binding less often, with a better overall performance in terms of deaths and output loss, especially in case of group testing. These results suggest that the benchmark results are actually lower bounds for the effective gains of contact tracing.

**Three-samples group testing**. In addition to the two-rounds group testing by Dorfman [17], I also considered an alternative with three samples per individual. First samples are bunched in groups of $\hat{g}$. In case of positive results, second samples are then bunched in subgroups of *g*, with $1\leq g\leq \hat{g}$ and such that $\hat{g}/g$ is integer. In case of a positive result for a subgroups, third samples are processed individually. The expected number of tests, in this case, is equal to:

$$nG_{3}(p,g,\hat{g})=\frac{n}{\hat{g}}\left\{\left(1-p{)}^{\hat{g}}+[1-(1-p{)}^{\hat{g}}][1+\frac{\hat{g}}{g}\;G(p,g)\right]\right\}$$ (22)

where *G*(*p*,*g*) is defined in equation 9. The optimal group sizes $\hat{g}$ and *g* are the result of the minimization of the expected number of tests. The testing capacity multiplier associated with this three-samples strategy is $1/G_{3}^{*}(p)$, where $nG_{3}^{*}(p)$ is the number of required tests at the optimal choices of $\hat{g}$ and *g*. As in the previous group testing simulation, I considered a simple rule-of-thumb forecast model for the positives to tests ratio. The results are summarized again in table 2. The gains from this strategy are actually very similar to the baseline, two-rounds, alternative. Most likely, the reason is that the number of tests grows much more rapidly, with respect to the two-samples case, when the ex-post ratio of positives to tests is bigger than the forecast, which often happens before the peak, when infections accelerate. Since the costs of collecting three samples is arguably higher, both for the laboratories and for the tested individuals, the conclusion is that two samples are sufficient.

### Robustness

Table 3 summarizes the performances of contact tracing, with or without group testing, for alternative parameter values. The goal of this exercise is twofold: to assess the robustness of the numerical results and to characterize the epidemics that could be successfully mitigated with those testing strategies. In all cases, I keep the two important assumptions that motivate the analysis, namely that the pathogen responsible for the epidemic can trigger asymptomatic infections and that the symptoms, whenever arise, are not specific, meaning that there exists more than one disease compatible with the same clinical signs. Without asymptomatic infections, or if symptoms could not be mistaken, than it would be optimal, for mitigation, to simply quarantine all of the symptomatics.

**Table 3:** Contact Tracing with Group Testing, Robustness.

	Test	Infpeak	Hospeak	Deaths	Yloss	Discovered
π=0.06, π=0.03	Hos	50.04	2.36	3.91	-4.95	32.46
	Trace	49.27	2.43	3.77	-5.64	49.47
	Trace+Group	49.17	2.33	3.81	-5.34	37.75
η ¯ =0.01	Hos	28.91	1.73	3.46	-4.03	32.49
	Trace	26.38	1.69	3.44	-4.74	43.93
	Trace+Group	24.46	1.63	3.25	-4.72	41.96
ρ=0.5	Hos	17.65	1.49	2.95	-3.32	32.18
	Trace	14.32	1.39	2.76	-3.47	46.78
	Trace+Group	11.23	1.27	1.94	-2.77	51.06
κ=0.15	Hos	15.68	1.87	2.53	-2.62	30.15
	Trace	10.32	1.74	1.68	-2.20	52.43
	Trace+Group	6.26	1.63	1.09	-1.54	62.31
β=0.1	Hos	14.25	2.26	2.07	-2.62	43.67
	Trace	10.24	2.06	2.04	-2.46	50.18
	Trace+Group	3.99	1.55	0.58	-0.85	67.79
γ=0.25	Hos	1.59	0.91	0.08	-0.08	21.82
	Trace	0.97	0.88	0.04	-0.04	38.95
	Trace+Group	0.90	0.85	0.03	-0.04	37.16
δ=0.01	Hos	18.18	1.46	5.04	-4.19	27.17
	Trace	11.46	1.18	4.01	-3.60	45.66
	Trace+Group	6.77	1.05	2.09	-2.13	62.48
*W* = 0.9	Hos	20.65	1.51	3.11	-3.36	26.35
	Trace	13.64	1.30	2.58	-3.28	43.81
	Trace+Group	6.03	1.04	1.16	-1.91	61.39

**Notes**: Infpeak is the percentage of the population infected at the peak of the epidemic (peak actual prevalence). Hospeak if the maximum fraction of the population to access an hospital. Deaths is the mortality rate as a percentage of the population, computed at the end of the epidemic. Yloss is the cumulative output loss (total output gap). Discovered is median of the ratio of discovered infected to total infected during the epidemic. Test is the testing policy. Hos refers to tests to hospitalized agents only. Trace refers to contact tracing and tests to hospitalized agents. Trace+Group refers to group testing in two rounds (1 grouping) in addition to contact tracing and tests to hospitalized agents only. Average values of the distribution of the results over all simulation runs with an epidemic. The parameters used in the simulation are summarized in table 1. π and π ¯ are the infection probabilities from, respectively, symptomatics and asymptomatics. ρ and κ are probability of developing symptoms in case, respectively, of infection or no infection. β is the probability of hospitalization. γ is the recovery probability. δ is the death probability. η¯ is the pre-epidemic density. *W* is the labor force participation.

Table 3 highlights two important scenarios in which contact tracing with group testing will not work: if the contagion probabilities π and π¯ are high, and in socially dense economies, characterized by high values of η¯. In both cases, which are associated with a high reproduction number, the epidemic spreads rapidly, and too many tests are required to make contact tracing work, so congestion is not avoidable even using group testing. Moreover, group testing is less efficient because of a high positive to tests ratio and because the adaptive forecasts become inaccurate due to the high time-series volatility of the infections. In such scenarios, it is necessary to implement lockdowns to reduce contagions or to build additional testing capacity to make contact tracing work. Notice that, in case of a higher labor force participation, which also implies a bigger pathogen reproduction number, contact tracing actually still works quite well, because of the assumption of perfect tracing on the workplace.

Contact tracing with group testing, as highlighted in table 3, is still associated with significant gains in case of more aggressive pathogens that more often yield symptomatic infections (high ρ) or that more often deliver severe symptoms that require hospitalizations (high β). Similarly, it works well in case of a pathogen that triggers symptoms^17^ with a higher incidence in the non-infected population (high κ). The gains from contact tracing and group testing also increase with the death rate (high δ), meaning that they will be higher in case of a binding health system capacity constraint that makes it impossible to simultaneously treat a big number of symptomatics, or in case of a higher median age of the population. In case of a high recovery rate (higher γ), the scope of the testing policies is more limited, since the aggregate consequences of the disease are less severe, but contact tracing with group testing still delivers sizable gains.

I also analyzed the robustness of the main results under alternative assumptions regarding voluntary tests. A potential reason why they yield sub-optimal results could be the high value of *Q* in the benchmark simulation, that translates into an insufficient number of agents that want to take tests even in case of a high observed disease prevalence and, therefore, into a mild endogenous mitigation. I simulated the model under a rather extreme alternative parametrization, in which 50% of the asymptomatics, and almost all of the symptomatics, want to get tested in case of a 5% observed prevalence, which implies *Q* = 0.08. The simulation results that were not different from the benchmark, with voluntary testing that is still a dominated policy.

Coming back to group testing, the rule-of-thumb forecast used to set the group size in case of group is not optimal and it is, in principle, possible for group testing to yield even bigger gains in case of a more sophisticated forecast model, although, at early stages of the epidemic, the lack of a sufficient time series of data impairs the forecast ability. I considered a further adaptive forecast model based on a simple rolling regression, according to which the forecast in *t* is based on information on the evolution of the epidemic up to date *t*–1. I included four variables in the regression: the lag of the positives to test ratio, the number of screened individuals, the stock of discovered infected and the fraction of the population which is hospitalized. The results turned out to be very similar to the rule-of-thumb alternative, with a slightly lower median percentage of discovered infected and a slightly smaller death rate. Actually this forecast model is also rudimentary, and this simulation results do not exclude the possibility that more sophisticated forecasts such as, for instance, the ones based on deep learning algorithms, might deliver better results. Once again, given the substantial implementation difficulties of complicated forecasts, it seems to be enough to focus on a simple rule-of-thumb, unless the pathogens has a high basic reproduction number (see above), in which case contact tracing with adaptive forecasts does not work well.

## Conclusion

Epidemics such as the one caused by Covid-19 are disruptive events, whose adverse negative consequences on the economy, and on society in general, can be enormous. Devising appropriate mitigation policies to curb those effects is therefore essential. Particularly important is an appropriate testing strategy to identify and isolate the infected, in order to stem contagions. Using a calibrated SIR model with production and endogenous responses to the epidemic, I showed that the optimal testing policy that minimizes a social loss function is contact tracing with group testing, consisting in identifying, and testing, the social contacts of the discovered infected individuals, pooling test in groups, and performing individual tests only in case of a positive result. This policy can smooth the peak of infections, ease the pressure on hospitals, reduce the mortality rate and curb the output loss, unless in case of: very contagious diseases; socially dense countries; very small testing capacity, that is a small maximum number of tests that the labs can process; high test costs. In case of a limited testing capacity, or in case of expensive tests, it is instead best to perform tests only on those individuals in critical conditions. The model also implies that, when tracing the social contacts of the infected, investing in complicated, costly, and privacy-invading tracing technologies is not worthwhile unless in case of a very large testing capacity. Finally, I showed that, when doing contact tracing, it is not recommended to also allow for voluntary tests. Summarizing, the policy conclusion is that contact tracing with group testing can significantly curb the negative effects of a pandemic for many diseases and economies.

## Footnotes

^1^ Contact tracing is also labeled TTQ: Testing-Tracing-Quarantine

^2^ Allowing a division of schools and universities into classes results in a smaller probability of getting the infection at school and it would increase the importance of social activities and family relationships as a source of infections. For fixed model parameters, it will also result in a slower progression of the epidemic.

^3^ 3 An alternative way for the agents to reduce the risk to which they are exposed is to adopt, for a fixe number of social matchings, precautionary measures that decrease the contagion probability, such as wearing a face mask for respiratory pathogens.The alternative model with an exogenous reduction of π and π proportionately to the observed disease prevalence is actually equivalent to the endogenous reduction of social contacts.

^4^ This is also the reason why a lockdown might be necessary even in case of a voluntary reduction of social activities by the agents, as in Eichenbaum, Rebelo and Trabandt [18].

^5^ I assume that it is possible to develop the same symptoms, in a given period, both because of the disease and because of the concurrent infection, and that, in such cases, the severity of the symptoms does not change.

^6^ The alternative model with a perceived risk that increases with social matchings will actually imply a smaller number of tests when the observed disease prevalence is high, thereby impairing the performance of voluntary tests. Thus I will evaluate the performance of voluntary testing in a best-case scenario.

^7^ The alternative is to assume that all agents behave as second infections were impossible, which means assuming no voluntary tests for the immunes. In this setting there will be less lab congestion and, in principle, a better performance of this strategy. However the testing capacity constraint is seldom binding in case of voluntary tests for the benchmark model parametrization, so the differences in performance will be, if anything, minimal.

^8^ The family members of the discovered infected agents are required to take a test, as part of contact tracing, only right after the discovery of the infection in the family. Infections that take place in subsequent periods, as a result ofrepeated family contacts that are not discontinued, can be therefore overlooked. There is therefore a limit to the percentage of the infected that can be discovered through contact tracing. The alternative would be to frequently test the family members of an infected, but this will also impair the performance of contact tracing by creating congestion.

^9^ This statement assumes that bunching samples together does not result in a ˝dilution˝ that makes it more complicated to detect the pathogen responsible for the contagious disease. In such cases, the group size must be reduced in order to restore the test sensitivity.

^10^ This testing strategy is different from the bunching of tests proposed by Jonnerby et al. [30] and that requires a quarantine for all members of groups that tested positive, which can be highly costly for the economy

^11^ This excludes the possibility that clusters of individuals who were in contact with each other are tested in the same session or labs. So I assume, unrealistically, that the samples in those clusters are processed in different labs.

^12^ The calibration is also similar to the one that I propose in a companion paper [37] that I use to study the effects of lockdowns

^13^ For small values of θ*i*, the number of social matchings decreases rapidly towards zero and it is very small even at relatively low values of the observed disease prevalence, while there is virtually no reduction at all when θ*i* is bigger than 10.

^14^ Asking the model to match, say, an increase in the observed virus prevalence without knowing exactly if the transmission probability increased because of the greater diffusion of a more aggressive strain or because the ban of voluntary tests was canceled or because the labs changed their priority policy results in too many degrees of freedom.

^15^ The starting point of the polices influences their performance. Implementing contact tracing soon enoughmight effectively stem the epidemic before it fully develops, rapidly bringing contagions to zero. Viceversa, when done too late, itmight be ineffective. For the baseline parameters, the epidemics last, on average, for 120 periods, so 10 periods seems a sensible choice, assuming that the authorities need time to organize. I obtained similar results in a small neighborhood of 10, between 8 and 12 periods since first infections.

^16^ In section , I also show that this congestion is not the by-product of the assumption that previously discovered infected agents are required to take tests for contact tracing.

^17^ In the Covid–19 example, these scenario can be thought as the summer, when a respiratory pathology is unlikely, while the baseline simulation refers to winter months, when Influenza typically strikes.
